# SGLT1 activity in lung alveolar cells of diabetic rats modulates airway surface liquid glucose concentration and bacterial proliferation

**DOI:** 10.1038/srep21752

**Published:** 2016-02-23

**Authors:** Tales Lyra Oliveira, Návylla Candeia-Medeiros, Polliane M. Cavalcante-Araújo, Igor Santana Melo, Elaine Fávaro-Pípi, Luciana Alves Fátima, Antônio Augusto Rocha, Luiz Ricardo Goulart, Ubiratan Fabres Machado, Ruy R. Campos, Robinson Sabino-Silva

**Affiliations:** 1 Institute of Biological Sciences and Health, Federal University of Alagoas, Alagoas, Brazil; 2 Department of Physiology, Federal University of São Paulo, São Paulo, Brazil; 3 National Reference Center of Leprosy and Sanitary Dermatology, Federal University of Uberlandia, Minas Gerais, Brazil; 4 Department of Physiology, Institute of Biomedical Sciences, University of Sao Paulo, Sao Paulo, Brazil; 5 Institute of Genetics and Biochemistry, Federal University of Uberlandia, Minas Gerais, Brazil; 6 Department of Medical Microbiology and Immunology, University of California Davis, California, USA; 7 Department of Physiology, Institute of Biomedical Sciences, Federal University of Uberlandia, Minas Gerais, Brazil

## Abstract

High glucose concentration in the airway surface liquid (ASL) is an important feature of diabetes that predisposes to respiratory infections. We investigated the role of alveolar epithelial SGLT1 activity on ASL glucose concentration and bacterial proliferation. Non-diabetic and diabetic rats were intranasally treated with saline, isoproterenol (to increase SGLT1 activity) or phlorizin (to decrease SGLT1 activity); 2 hours later, glucose concentration and bacterial proliferation (methicillin-resistant *Sthaphylococcus aureus*, MRSA and *Pseudomonas aeruginosa, P. aeruginosa*) were analyzed in bronchoalveolar lavage (BAL); and alveolar SGLT1 was analyzed by immunohistochemistry. BAL glucose concentration and bacterial proliferation increased in diabetic animals: isoproterenol stimulated SGLT1 migration to luminal membrane, and reduced (50%) the BAL glucose concentration; whereas phlorizin increased the BAL glucose concentration (100%). These regulations were accompanied by parallel changes of *in vitro* MRSA and *P. aeruginosa* proliferation in BAL (r = 0.9651 and r = 0.9613, respectively, Pearson correlation). The same regulations were observed in *in vivo P. aeruginosa* proliferation. In summary, the results indicate a relationship among SGLT1 activity, ASL glucose concentration and pulmonary bacterial proliferation. Besides, the study highlights that, in situations of pulmonary infection risk, such as in diabetic subjects, increased SGLT1 activity may prevent bacterial proliferation whereas decreased SGLT1 activity can exacerbate it.

The luminal surface of airway epithelium is covered by a thin layer of fluid, termed the airway surface liquid (ASL)^1^,^2^. The volume and composition of ASL are carefully regulated and play an important role in lung defense^3^. ASL glucose concentration is 3–20 times lower in ASL than in plasma^4^, and results from the balance of epithelial glucose efflux and influx. Glucose flows from the interstitium to the ASL down its concentration gradient, through a paracellular pathway, in both proximal (trachea, bronchi and bronchioles) and distal (alveolar) lung^5^. On the other hand, ASL glucose uptake takes place in luminal membrane through the GLUT2-mediated facilitative glucose diffusion in proximal airways; and through the SGLT1-mediated sodium-dependent glucose transport in the distal lung^6^. It has been speculated that GLUT2-mediated glucose reabsorption depends on a concentration gradient generated by intracellular glucose metabolism^1^,^7^,^8^ and, probably, basolateral efflux does not occur at this proximal segment, since a glucose concentration gradient is unexpected. Differently, in the distal lung epithelium, glucose is transported against it gradient concentration, accumulates in the intracellular and thus can efflux into the interstitium by a facilitative transport^9^. Additionally, the *SLC5A1* mRNA has also been identified in distal lung epithelium of animal and human epithelium^1^,^10^,^11^,^12^. The SGLT1 protein has been described on the surface of type I^13^ and type II^14^ pneumocytes. The instillation of phlorizin, an inhibitor of SGLT1 cotransporter, decreased glucose reabsorption in rat lung *in situ* under normoglycemic conditions^15^, suggesting an important role of SGLT1 in ASL glucose homeostasis.

Hyperglycemia in diabetic patients is associated with elevated prevalence of respiratory complications^16^, and predisposes the host to bacterial infections^1^. The presence of high levels of glucose in ASL could predispose to respiratory infection through direct effects on bacterial growth^1^,^6^,^17^. Multiple respiratory pathogens such as methicillin-resistant *Staphilococcos aureus* (MRSA) and *Pseudomonas aeruginosa* (*P. aeruginosa*) are isolated more frequently from sputum of hyperglycemic critically ill patients, and that has been associated with increased glucose concentration in ASL^3^,^18^,^19^. Despite the direct effect of hyperglycemia on ASL glucose concentration, and consequently on bacterial proliferation, the capacity of the SGLT1 transporter to modulate these effects has never been investigated in diabetic condition.

The cAMP-PKA pathway mediates important effects of the non-selective beta-adrenergic agonist isoproterenol on pulmonary functions^20^. In SGLT1-transfected Xenopus laevis oocytes, as well as in epithelial cells of intestine and salivary glands, it has been clearly demonstrated that the activation of cAMP-PKA pathway enhances the SGLT1-mediated glucose transport, and that is related to the increased translocation of SGLT1 protein from the cytoplasm to the plasma membrane^21^,^22^,^23^. Because of that, we hypothesize that cAMP-PKA-mediated beta-adrenergic activity, induced by isoproterenol, might also increase ASL glucose reabsorption via SGLT1 in pneumocytes of distal lung.

Thus, the aims of the present study were to investigate: 1) the SGLT1 protein subcellular localization in alveolar cells; 2) the glucose concentration on bronchoalveolar lavage (BAL); 3) the proliferation of MRSA and *P. aeruginosa* on BAL, in lung from diabetic rats acutely treated (2 hours after intranasal infusion) with isoproterenol or phlorizin. Our findings related to the SGLT1 activity in the alveolar epithelium of diabetic rats open new perspectives for the development of drugs that can minimize or maximize respiratory infections, arising from regulation of glucose concentration in ASL.

## Results

As proposed, non-diabetic (ND) and diabetic (D) rats were acutely treated with saline (s), isoproterenol (i), and phlorizin (p); thus, the following groups were studied: NDs, NDi, NDp, Ds, Di and Dp.

To confirm the effectiveness of intranasal isoproterenol treatment, hemodynamic parameters were assessed in animals anesthetized with sodium thiopental (Supplementary Figure 1). The results show that, 15 min after intranasal isoproterenol, there was an increase (*P* < 0.05) in mean arterial pressure and heart rate. These increases disappeared after ~70 min, evincing that when the lung or BAL were sampled (120 min), the cardiovascular effects of isoproterenol had already dissipated. Phlorizin treatment effectiveness was confirmed by alterations in ASL glucose concentration, as presented below.

**Figure 1:**
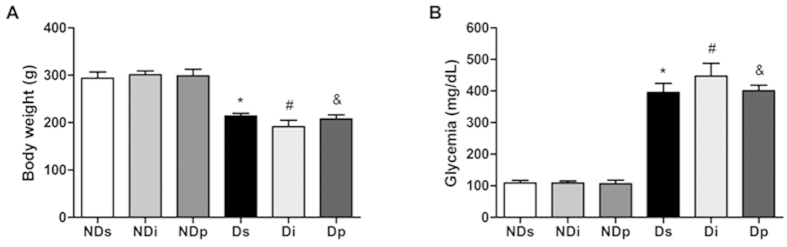
General parameters. Body weight **(A)** and blood glucose concentration **(B)** of non-diabetic saline (NDs), non-diabetic isoproterenol (NDi), non-diabetic phlorizin (NDp), diabetic saline (Ds), diabetic isoproterenol (Di) and diabetic phlorizin (Dp) treated rats. Results are mean ± SEM of 6–8 animals; ^*^P < 0.05 vs NDs, #*P* < 0.05 vs NDi, and &*P* < 0.05 vs NDp; one-way ANOVA followed by Student Newman Keuls post-test.

Figure 1 shows that, as expected, diabetes reduced body weight (*P* < 0.05) and increased blood glucose levels (*P* < 0.05). Treatment of neither isoproterenol nor phlorizin altered these parameters.

### Diabetes, isoproterenol and phlorizin do not change alveolar structures in rats

**Figure 2:**
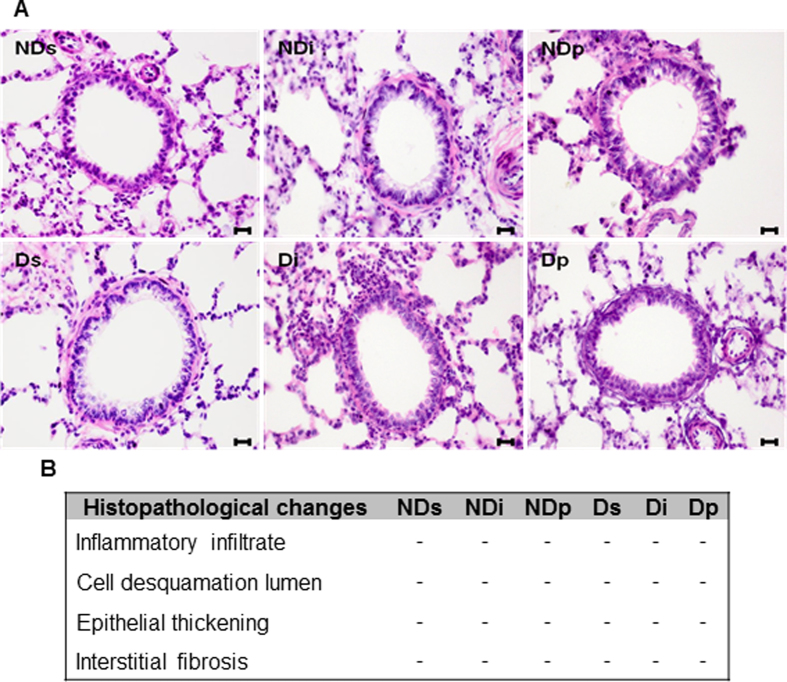
Hematoxylin-eosin stained of lung tissue. Alveolar and bronchiolar structures in lung from non-diabetic saline (NDs), non-diabetic isoproterenol (NDi), non-diabetic phlorizin (NDp), diabetic saline (Ds), diabetic isoproterenol (Di) and diabetic phlorizin (Dp) treated rats. Hematoxylin-eosin stained sections (**A**), magnification, x400, scale bar, 20 μm, and potential histopathological alterations **(B)**; present (+) and absent (−). Images are representative of 4–6 animals in each group.

To determine whether diabetes, isoproterenol and phlorizin promoted morphostructural changes in lung, we stained the lung sections with hematoxylin-eosin. The alveolar and bronchiolar structures remained unaltered (Fig. 2A). Besides, treatments did not promote changes in inflammatory infiltrate, cell desquamation in lumen, epithelial thickening and interstitial fibrosis (Fig. 2B).

### Diabetes, isoproterenol and phlorizin modulate mucus secretion

**Figure 3:**
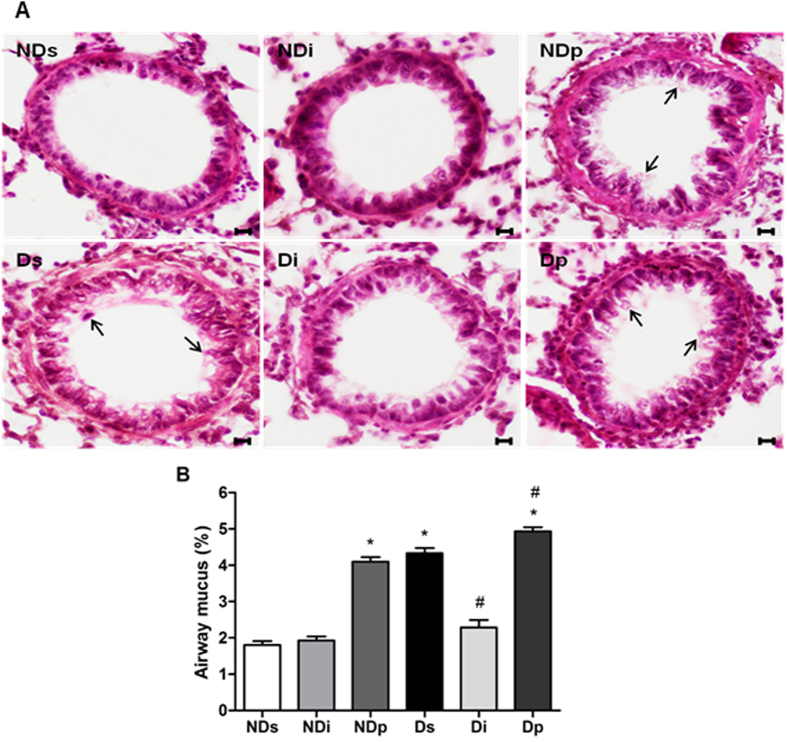
Periodic acid–Schiff (PAS) staining of lung tissue. Bronchiolar PAS stained sections of non-diabetic saline (NDs), non-diabetic isoproterenol (NDi), non-diabetic phlorizin (NDp), diabetic saline (Ds), diabetic isoproterenol (Di) and diabetic phlorizin (Dp) treated rats. (**A**) Photomicrographs of PAS stained lung; black arrows indicate the pink-magenta stained mucus in bronchiolar lumen; magnification, ×400, scale bar, 20 μm. (**B**) Quantitative mucus production, expressed as mucus filled area related to total airway lumen area. Results are mean ± SEM of 4–6 animals; ^*^P < 0.05 vs NDs; and ^#^*P* < 0.05 vs Ds. One-way ANOVA followed by Student Newman Keuls post-test for mean comparisons.

Mucus secretion in bronchiolar lumen is shown in Fig. 3. NDs, NDi and Di rats showed low mucus content; whereas the mucus content is clearly increased (Fig. 3A) in the other groups. The quantitative analysis (Fig. 3B) of PAS stained area related to the airway luminal area confirmed that diabetes increased mucus secretion (*P* < 0.05). Besides, the mucus content decreased after isoproterenol treatment in diabetic rats (Di vs Ds, *P* < 0.05), whereas it increased after phlorizin treatment in non-diabetic (NDp vs NDs, *P* < 0.05) and diabetic rats (Dp vs Ds, *P* < 0.05).

### Subcellular distribution of SGLT1 protein in alveolar cells

**Figure 4:**
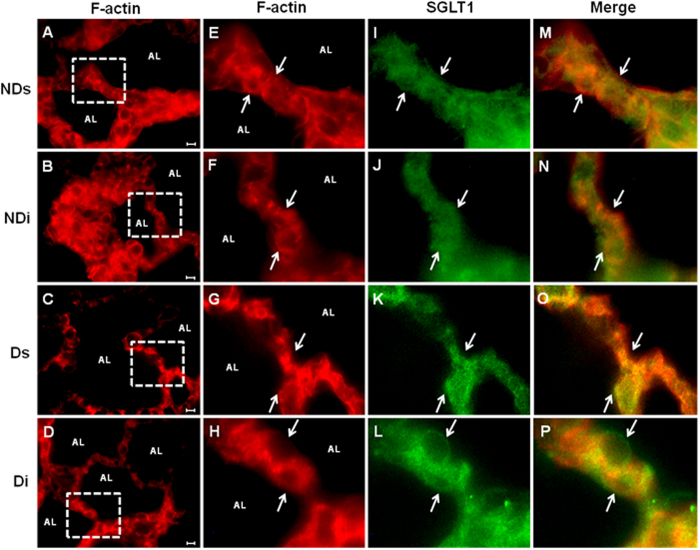
Immunodetection of SGLT1 protein in a section of rat pulmonary alveoli. Section of pulmonary alveoli from non-diabetic saline (NDs), non-diabetic isoproterenol (NDi), diabetic saline (Ds) and diabetic isoproterenol (Di) treated rats. Sections (**A–D)** were immunostained with anti-F-actin antibody (red). Enclosed boxes showing an alveolar septum, taken with a greater resolution, are presented in the next sections: **(E**–**H)** F-Actin (red), **(I**–**L)** SGLT1 (green) and **(M–P)** merged photomicrographs for colocalization of SGLT1 and F-actin (yellow to orange). White arrows indicate the presence of SGLT1 in the luminal membrane. Description: (AL) alveolar lumen of the rat pulmonary section. Magnification, ×1000, scale bar, 10 μm. Images are representative of 4–5 animals in each group.

**Figure 5:**
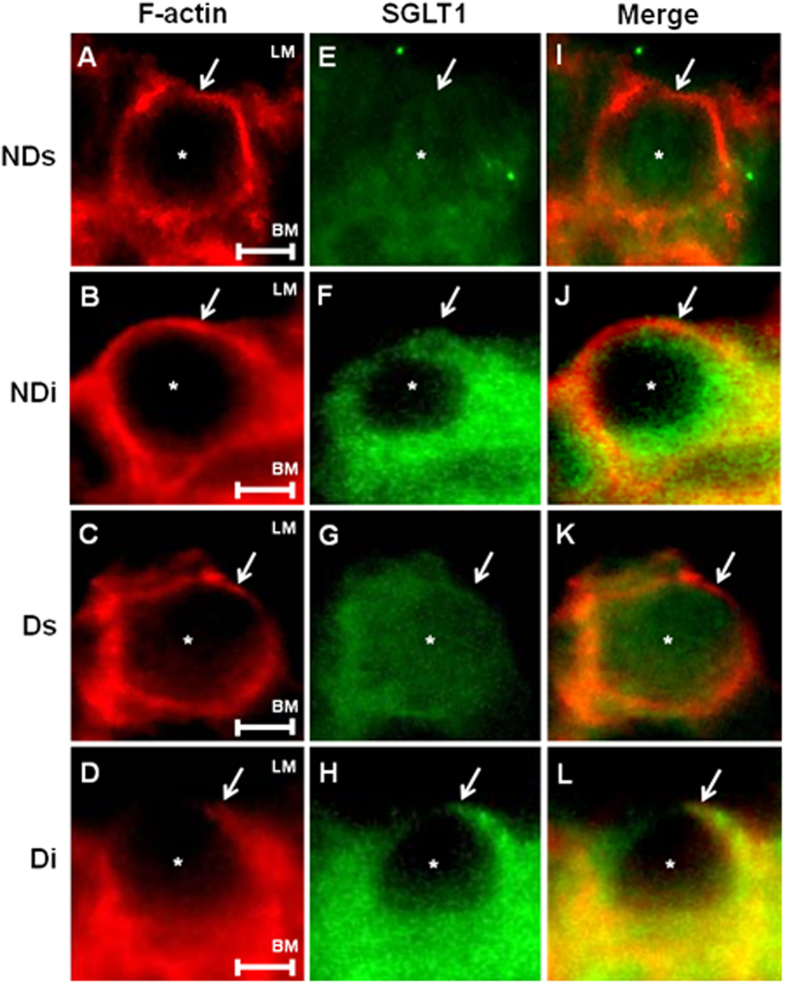
Immunodetection of SGLT1 protein in lung alveolar cells. SGLT1 protein in lung alveolar cells from non-diabetic saline (NDs), non-diabetic isoproterenol (NDi), diabetic saline (Ds) and diabetic isoproterenol (Di) treated rats. **(A**–**D)** F-actin (red), **(G,H)** SGLT1 (green) and **(I**–**L)** merged photomicrographs for colocalization of SGLT1 and F-actin. White arrows indicate the presence of SGLT1 in the luminal membrane, and the asterisks indicate the presence of SGLT1 in the cytoplasm. Description: (LM) luminal membrane and (BM) basolateral membrane of the pneumocyte. Magnification, ×1000, scale bar, 2 μm. Images are representative of 4–5 animals in each group.

Immunodetection of SGLT1 (green color) and F-actin (red color) in sections of rat pulmonary alveoli and in isolated alveolar cell is shown in Figs 4 and 5, respectively. Images from phlorizin-treated rats are presented as Supplementary Figure 2, since SGLT1 immunodetection after this treatment became almost imperceptible; thus, adding no relevant data.

Figure 4 shows the F-actin immunodetection in sections of pulmonary alveoli (4A to 4D), from which the squared marked alveolar septum was amplified and analyzed for F-actin (4E to 4H) and SGLT1 (4I to 4L), as well as for the merged image (orange to yellow colors, 4M to 4P). The SGLT1 was detected in the intracellular region (reserve pool) and in the luminal membrane as well. Merged images from isoproterol-treated NDi and Di rats depict yellow color as well as an outlined green color (4N and 4P) at the luminal region, suggesting increased SGLT1 translocation to the plasma membrane.

Figure 5 shows the immunoreactivity for F-actin (5A to 5D) and SGLT1 protein (5E to 5H), as well as the merged image (5I to 5L), in isolated alveolar cells of lung. In cells from NDs (5E) and Ds (5G) rats, SGLT1 protein is mainly detected in the intracellular region. The isoproterenol treatment (5F and 5H) promoted a strong reduction in the intracellular SGLT1 content, and that was accompanied by enhanced expression of SGLT1 in plasma membrane including the luminal surface. The coexpression of SGLT1 and F-actin, as evinced by the yellow color, reinforced the isoproterenol-induced SGLT1 mobilization from intracellular to plasma membrane (5J and 5L). No evident effects of diabetes on SGLT1 expression or subcellular distribution were observed.

### Effect of isoproterenol and phlorizin on BAL glucose concentration of diabetic rats

**Figure 6:**
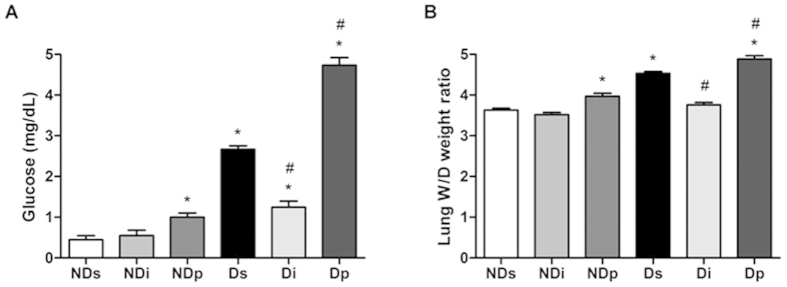
Bronchoalveolar lavage glucose concentration and lung water content. Bronchoalveolar lavage glucose concentration **(A)** and lung water content **(B)** were analyzed in samples from non-diabetic saline (NDs), non-diabetic isoproterenol (NDi), non-diabetic phlorizin (NDp), diabetic saline (Ds), diabetic isoproterenol (Di) and diabetic phlorizin (Dp) treated rats. Lung water content was estimated from the wet (W)/dry (D) tissue weight ratio. Results are mean ± SEM of 4–7 animals; ^*^P < 0.05 vs NDs; ^#^*P* < 0.05 vs Ds. One-way ANOVA followed by Student Newman Keuls post-test.

Figure 6A shows the glucose concentration measured in BAL. Diabetes significantly increased the BAL glucose (Ds vs NDs, *P* < 0.05). Phlorizin treatment increased (*P* < 0.05) the BAL glucose in both non-diabetic and diabetic rats. Differently, isoproterenol treatment decreased the BAL glucose content in diabetic rats (*P* < 0.05), but had no effect on non-diabetic rats, in which the glucose concentration was already very low. The lung water content was also analyzed based on the measurement of the tissue wet/dry weight ratio; regulations similar to those observed in BAL glucose were observed (Fig. 6B).

### Effects of isoproterenol and phlorizin on MRSA and *P. aeruginosa* proliferation in BAL of diabetic rats

**Figure 7:**
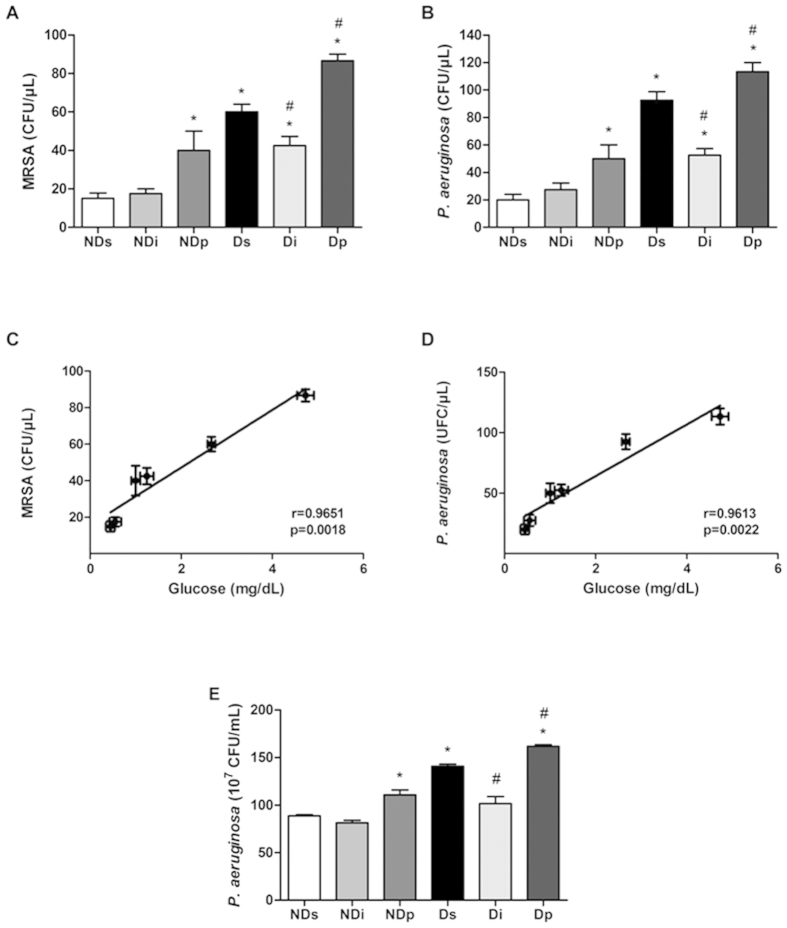
Bacterial proliferation analysis in bronchoalveolar lavage and lung tissue. Bacterial proliferation was analyzed in samples from non-diabetic saline (NDs), non-diabetic isoproterenol (NDi), non-diabetic phlorizin (NDp), diabetic saline (Ds), diabetic isoproterenol (Di) and diabetic phlorizin (Dp) rats. *In vitro* proliferation of methicillin-resistant *Staphylococcus aureus* (MRSA, panel **A**) and *Pseudomonas aeruginosa* (*P. aeruginosa*, panel **B**) were analyzed in bronchoalveolar lavage samples (BAL). Mean values of BAL glucose concentration (from Fig. 6) and of bacterial proliferation rate were analyzed by Pearson correlation test (panels **C** and **D**). *In vivo P. aeruginosa* proliferation was analyzed in lung tissue samples collected 6 hours after bacterial inoculation (panel **E**). Results are mean ± SEM of 4–6 animals; ^*^P < 0.05 vs NDs, ^#^*P* < 0.05 vs Ds; one-way ANOVA followed by Student Newman Keuls post-test.

To our knowledge, this is the first time that the proliferation of MRSA (Fig. 7A) and *P. aeruginosa* (Fig. 7B) in BAL of diabetic rats under intranasal treatment with saline, isoproterenol or phlorizin is described. In non-diabetic rats, isoproterenol treatment did not alter (*P* > 0.05) bacterial proliferation rate; however, phlorizin treatment increased (*P* < 0.05) the proliferation rate of MRSA and *P. aeruginosa*. Diabetes increased the bacterial proliferation rate (Ds vs NDs, *P* < 0.05); and, in diabetic rats, isoproterenol treatment decreased (*P* < 0.05) whereas phlorizin treatment increased (*P* < 0.05) the bacterial proliferation of both MRSA and *P. aeruginosa*. Importantly, the Pearson correlation analysis shows that the mean values of MRSA (Fig. 7A) and *P. aeruginosa* (Fig. 7B) proliferation rates and the respective means values of BAL glucose concentration correlated positively (r = 0.9651 and r = 0.9613, respectively) and significantly (*P* < 0.05).

In order to evaluate whether these effects also occur *in vivo*, *P. aeruginosa* proliferation was analyzed in a homogenate of a whole pulmonary tissue sampled 6 hours after bacterial inoculation, with the same previous saline, isoproterenol and phlorizin treatments being applied 1 hour before inoculation and again reinforced 1 hour before euthanasia (Fig. 7C). The *P. aeruginosa* proliferation rate profile was exactly the same of that observed when *P. aeruginosa* was added to the BAL *in vitro* (Fig. 7B).

## Discussion

Depletion of ASL glucose is fundamental to guarantee the airway sterility in lung, and might prevent microbial infection in diabetic patients^1^. Modulation of the Na^+^-glucose coupled carrier SGLT1 activity, altering the ASL glucose concentration and the risk of respiratory infections, has not been tested yet. We showed that improvement of SGLT1 activity by isoproterenol decreases ASL glucose concentration and microbial proliferation; and, conversely, repression of SGLT1 activity by phlorizin increases ASL glucose concentration and microbial proliferation as well, in lung of diabetic rats.

Apparently, diabetes did not alter the SGLT1 content in pulmonary alveolar cells. However, the pre-treatment with isoproterenol clearly reduced the intracellular SGLT1 content, increasing its translocation to the luminal membrane; although not exclusively. Participation of β-adrenergic activity on subcellular SGLT1 localization has already been described in intestinal cells^24^, and in acinar and ductal cells of salivary glands^23^,^25^. For the first time, we here demonstrate the isoproterenol-induced SGLT1 migration from the intracellular to the luminal membrane of pulmonary alveolar cells. This finding predicts that isoproterenol, by increasing the SGLT1-mediated alveolar glucose re-uptake, can reduce the ASL glucose concentration.

Surprisingly, the pre-treatment with phlorizin virtually blocked all SGLT1 immunoreactivity mainly at the cell surface (Supplementary Figure 2). Certainly this result can not represent a 2-hour induced disappearance of the transporter. Regarding that, we point out recent studies that have characterized the molecular bases of the phlorizin binding domain in SGLT1 molecule^26^. Phlorizin binds at the 13^rd^ transmembrane segment of SGLT1 (Phe602 and Phe609); additionally, its sugar moiety binds in the D-glucose binding site (Gln457 and Thr460)^27^, which is close to the SGLT1 antibody binding domain (457 to 460 sequence). Thus, we propose that the phlorizin binding into SGLT1 should prevent the subsequent binding of the SGLT1 antibody, explaining the weakening of SGLT1 immunoreactivity in phlorizin-treated animals. However, it is important to highlight that the blocking of the glucose transport throughout SGLT1 (our goal) was certainly guaranteed, regardless of its immunodetection.

Glucose concentration in ASL is closely related to plasma glucose concentration in normoglycemic and hyperglycemic conditions^3^. According to the glucose concentration gradient, glucose diffuses from plasma to ASL through paracellular pathway, and that is increased under hyperglycemic condition^1^,^3^. Apparently, the SGLT1 expression at the luminal membrane of alveolar cells of diabetic rats was unchanged; thus explaining why ASL glucose concentration increases: the increased plasma to ASL glucose flux is not counterbalanced by increased SGLT1-mediated re-uptake. According to that, in diabetic rats, when isoproterenol reduced the SGLT1 intracellular and increased its expression at the luminal membrane of alveolar cells, decreased glucose concentration in BAL was detected. On the other hand, the decrease of SGLT1 function in luminal membrane of alveolar cells after phlorizin treatment increased glucose concentration in BAL. Thus, the inversely parallel regulation of luminal SGLT1 function and glucose concentration of ASL points out that this transporter can be considered a new potential target for respiratory infection associated to higher levels of glucose in airway secretions.

Considering the capacity of SGLT1 to transport 264 molecules of water during the Na^+^/glucose transport cycle^28^,^29^,^30^, the increase in this protein at the luminal membrane of alveolar cells observed in isoproterenol-treated diabetic rats might be related to the decreased pulmonary water content verified in these animals; as well as the converse results observed in phlorizin-treated rats. Thus, the inversely parallel regulation of luminal SGLT1 activity and pulmonary water content indicates that this transporter may also be involved in the regulation of ASL volume. Besides, comparing to the water pulmonary content, parallel regulations were observed in the bronchiolar mucus content, indicating an additional mechanism related to the total water pulmonary regulation. Yet, the diabetes-induced increase in total water content must include the osmotic effect of high interstitial glucose concentration, regardless of the mucus and ASL volume regulations.

Taking into account the considerations above, we are proposing that the SGLT1 activity as a fundamental modulator of the ASL glucose concentration in hyperglycemic states. Despite that, other potential involved mechanisms should be discussed. The facilitative glucose transporter GLUT2 participation should be ruled out, since this transporter has never been described in native lung alveolar cells yet^7^. Similarly, locally isoproterenol-induced changes in blood perfusion should also be ruled out, since its cardiovascular effects were transient, and did not alter blood glycemia, as showed in Supplementary Figure 1. Finally, participation of luminal mucus and/or ASL volumes needs to be disregarded in the BAL glucose regulation, since their regulations occurred in parallel. For instance, if the isoproterenol-induced decrease in mucus and/or water content were to alter glucose concentration, it should increase it, and not decrease it, as we observed.

ASL is normally sterile despite constant exposure to bacteria. The balance between bacterial growth and killing in ASL determines the outcome of exposure to inhaled or aspirated bacteria: infection or sterility^1^. Several studies have shown that acute hyperglycemia is associated with poor outcomes from hospital admission for pulmonary infection. Patients with nosocomial pneumonia who have blood glucose concentration >11 mmol/L show an increased risk of death and in-hospital complications compared with those with blood glucose concentration ≤11 mmol/L^18^,^31^,^32^. Despite all reports that high blood glucose levels^18^ and/or high glucose concentration in bronchial aspirates^31^ predispose to pulmonary infection, the demonstration that short-time induced increase or decrease in BAL glucose parallels bacterial proliferation in diabetes has never been reported. Our data show the high correlation between mean BAL glucose concentration and MRSA and *P. aeruginosa* proliferation just 2 hours after isoproterenol or phlorizin respiratory inhalation. Besides, *in vivo* 6-hour-induced proliferation of *P. aeruginosa* was similarly regulated. These data highlight the powerful role of the SGLT1 activity in modulating ASL glucose concentration, and, consequently, bacterial proliferation.

Interestingly, the present study is an alert to the potential risk of pulmonary infection in diabetic patients using SGLTs inhibitors to improve glycemic control. Rapidly, the pharmaceutical industry has supplied the market with SGLT2 inhibitors, as coadjuvant drugs for hyperglycemia treatment^33^. Besides, seeking to expanding their action mechanism, the dual SGLT2 and SGLT1 inhibitors, such as canagliflozin^34^ and LX4211^35^, have been introduced in the diabetes pharmacopeia. However, whether systemically induced SGLT1 inhibition in diabetic patients is increasing pulmonary infection, especially in-hospital hyperglycemic subjects, has never been considered.

To the best of our knowledge, this is the first report to reveal that SGLT1 protein activity of the lung alveolar cells regulates the glucose concentration in the airway surface liquid and, consequently, the pulmonary bacterial proliferation risk, in diabetic state. Furthermore, the study unravels effects of β-adrenergic stimulation, which paves the way for new therapies able to prevent or fight pulmonary bacterial infection in diabetic patients. Finally, the study contributes to revealing that in-hospital diabetic patients taking the dual SGLTs inhibitors are at very high risk of pulmonary infection, and drug withdrawal should be considered.

## Methods

All experimental procedures were approved and conducted by the Ethical Committee for Animal Research of the Federal University of Alagoas (Approval No. 40/2012) and Ethical Committee for Animal Research of the Federal University of Sao Paulo (Approval No. 1281300915). All experimental protocols were conducted in accordance with the approved guidelines. Male Wistar rats (weighing ~260 g) were rendered diabetic (D) by a single intravenous injection of alloxan (40 mg/Kg body weight), and non-diabetic rats (ND) were injected with saline^23^,^36^. Animals were individually caged and allowed free access to water and standard rodent chow diet (Nuvilab CR-1; Nuvital, Curitiba, Brazil). ND and D rats were studied 21 days after diabetes induction. To demonstrate the effects of sympathetic activity and the inhibition of SGLT, ND and D rats were subjected to saline (0.9%; 100 μL; NDs and Ds), isoproterenol (5 mg/kg; 100 μL; NDi and Di) or phlorizin (10^−3^ M; 100 μL; NDp and Dp) treatment 2 hours before sampling. The treatments were performed intranasally, using a micropipette, and under anesthesia (60 mg/kg sodium thiopental, intraperitoneally). Blood, bronchoalveolar lavage, and tissue samples were always obtained from anesthetized rats, and in accordance with the approved guidelines.

### Measurement of isoproterenol effects on the cardiovascular system

To confirm the effectiveness of isoproterenol treatment, non-diabetic rats were anesthetized with sodium thiopental (60 mg/kg, ip), and fitted with femoral arterial catheter. After at least 24 h of surgical recovery, mean arterial pressure (MAP) and heart rate (HR) were recorded in anesthetized rats, through an analog-digital board that communicated with PowerLab software (ADInstruments, Sydney, Australia). MAP and HR of ND and D rats were recorded for 10 minutes (baseline: time 0) and thereafter rats were treated with intranasal isoproterenol (5 mg/kg; 100 μL). Cardiovascular records were followed for 2 hours. Additionally, blood samples were collected to evaluate glucose concentration at times zero and 2 hours.

### Volume measurement of pulmonary water content

A lung fragment was removed, immediately weighed (wet-weight, W), placed in an oven at 40 °C for 24 hours, and reweighed (dry-weight; D). The W/D weight ratio was calculated as described previously^37^, and used to represent the total pulmonary water content^38^,^39^.

### Collection of bronchoalveolar lavage (BAL) and tissue sampling

Following anesthesia, the chest cavity was accessed, to expose the lung and the trachea. A 19-gauge needle was gently inserted into the trachea, to inject 1 mL of chilled saline; thereafter, gentle aspirations were performed to collect 500 μL of a bronchoalveolar lavage (BAL), which was immediately stored at −20 °C for further analysis. Finally, the left ventricle was sectioned, the lungs were exsanguinated by saline infusion throughout the right ventricle, and tissue samples were collected for further analysis.

### Hematoxylin-eosin and periodic acid-Schiff staining of lung samples

Parts of the left lung were fixed with 4% formaldehyde prior to paraffin embedding to preserve the pulmonary architecture. Tissue sections were deparaffinized in xylol (xylene) and rehydrated through a graded series of ethanol to distilled water. Tissues were cut into 5-μm sections for hematoxylin-eosin (HE) and periodic acid-Schiff staining (PAS) to evaluate histopathological profile and mucus production, respectively. Tissues were examined by light microscope (Olympus BX51 attached DP70 Digital Camera System). To quantify mucus production, ImageJ analyzer software (National Institutes of Health, Bethesda, MD USA) was used to calculate the percentage of the bronchiolar lumen area that was stained by PAS.

### Immunohistochemistry analysis

Lung tissues were fixed in 4% formaldehyde phosphate buffer (PB) followed by cryoprotection in increasing sucrose solutions (10%, 20% and 30%) in PB. Seven-μm-thick sections were placed on gelatin-coated slides (Sigma Chem. Co., St Louis, USA), and subjected to immunodetection using anti-rat SGLT1 antibody (1:100, Merck Milipore, Germany, catalog number 07–1417), followed by incubation with Alexa Fluor 488 (1:200, Molecular Probes, Eugene, Oregon, USA, catalog number A21441). Reaction controls consisted of non-inclusion of primary antibodies. F-actin staining was performed with rhodamin-phalloidin (1:100, Molecular Probes, Merck Milipore, Germany, catalog number R415). After washings, tissue sections were coverslipped and analyzed in a fluorescence microscope (Nikon Eclipse 50i). Images were recorded for further analysis using ImageJ Software (National Institutes of Health, Bethesda, MD USA). Selected images are representative of 4 animals in each group.

### *In vitro* bacterial proliferation

Bronchoalveolar lavage (BAL) collected 2 hours after isoproterenol or phorizin treatment was used for the analysis of the *in vitro* bacterial proliferation. Methicillin-resistant *Staphylococcus aureus* - MRSA (ATCC BAA 976-1) and *Pseudomonas aeruginosa* (ATCC 27853-1) (bioMérieux) aliquots of 10^3^ cells were mixed to 125 μL of BAL, and samples of 1 μL of the mixture were inoculated, in sterile conditions, in Petri dishes (90 mm) containing blood agar. Inoculation was performed in triplicate, in three different plates. The mean of the triplicate was considered as one sample, and the number of the samples (animals) is informed in the legend. The plates were incubated at 37 °C for 48 h. Bacterial cultures were quantified by specifying the number of colony forming units (CFU), where each CFU was equivalent to 10^3^ bacteria. The results are shown as CFU/μL^40^.

### *In vivo P. aeruginosa* proliferation

The bacterial inoculum used for pneumonia induction consisted of *P. aeruginosa* suspension (ATCC 27853) freshly prepared from an overnight blood agar culture. The optical density of the bacterial suspension was adjusted for 10 McFarland units (3.0 × 10^9^ CFU/mL), according to the turbidity index^41^. Non-diabetic and diabetic rats were anaesthetized with isofluorane (0.08 to 1.5%), and an endotracheal intubation was performed, to instill 100 μL of saline (0.9%), isoproterenol (5 mg/Kg) or phlorizin (10^−3^ M). After 1 h, the animals were again subjected to endotracheal intubation, under anesthesia, to instill 200 μL bolus of the bacterial inoculum^41^. Five hours (5 h) after bacterial inoculation, the animals were once more instilled with 100 uL of saline (0.9%), or isoproterenol (5 mg/Kg) or phlorizin (10^−3^ M). One hour after the last treatment (6 hours after the *P. aeruginosa* inoculation), the animals were anesthetized with a lethal dose of thiopental (200 mg/kg b.w., ip), the right lung was removed, homogenized in 10 mL of sterile saline, and 10-fold diluted. After that, aliquot of 100 μL sample was added to 900 μL of growth medium Luria-Bertani, and incubated under stirring at 37 °C for 22 h. Finally, the samples were analyzed by optical spectrophotometria (at 600 nm)^1^, and the results were converted to McFarland units, and finally expressed as CFU/mL.

### Analytical Procedures

Glucose concentration in BAL samples was measured using the COBAS Integra 400 (Roche Diagnostics GmbH, Mannheim), with the Glucose HK Gen.3 assay (Roche Diagnosis)^42^. Blood glucose concentration was measured by a glucometer (Precision QID, MediSense, Sao Paulo, SP, Brazil).

### Statistical analysis

All values are reported as mean ± SEM. Number of animals is informed in the legends. Comparisons of the means were performed by one-way analysis of variance (ANOVA), followed by mean comparisons through the Student-Newman-Keuls post-test (GraphPad Prism version 5.00 for Windows, GraphPad Software, San Diego, CA, USA). The correlation between mean values of BAL glucose concentration and *in vitro* bacterial proliferation rate were analyzed by the Pearson correlation test. Values of *P* < 0.05 were considered as statistically significant.

## Additional Information

**How to cite this article**: Oliveira, T. L. *et al.* SGLT1 activity in lung alveolar cells of diabetic rats modulates airway surface liquid glucose concentration and bacterial proliferation. *Sci. Rep.* **6**, 21752; doi: 10.1038/srep21752 (2016).

## Supplementary Material

Supplementary Information
