# Supplementary material for: SGLT1 activity in lung alveolar cells of diabetic rats modulates airway surface liquid glucose concentration and bacterial proliferation
Source: Sci Rep. 2016 Feb 23;6:21752. doi: 10.1038/srep21752 (PMC4763199; doi:10.1038/srep21752)
Supplement: Supplementary Information [file srep21752-s1.pdf]

Supplementary Figure 1

SGLT1 activity in lung alveolar cells of diabetic rats modulates airway surface liquid glucose concentration and bacterial proliferation

Tales Lyra Oliveira, Návylla Candeia Medeiros, Polliane M. Cavalcante-Araújo, Igor Santana Melo, Elaine Fávaro-Pípi, Luciana Alves-Fátima, Antônio Augusto Rocha, Luiz Ricardo Goulart Filho, Ubiratan Fabres Machado, Ruy R Campos, Robinson Sabino-Silva

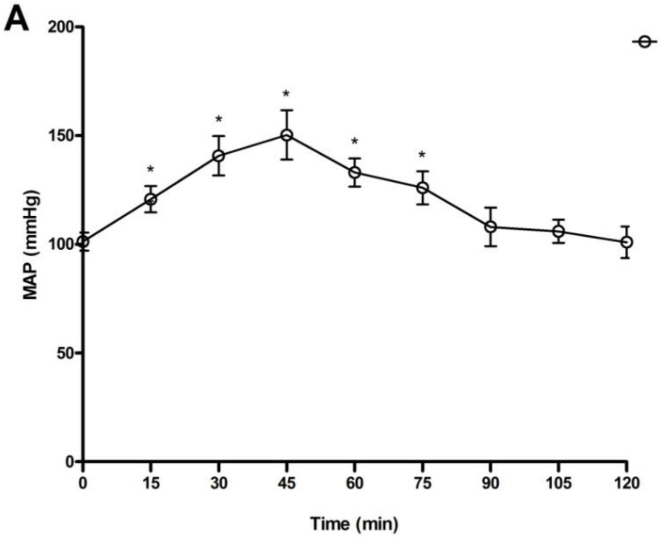

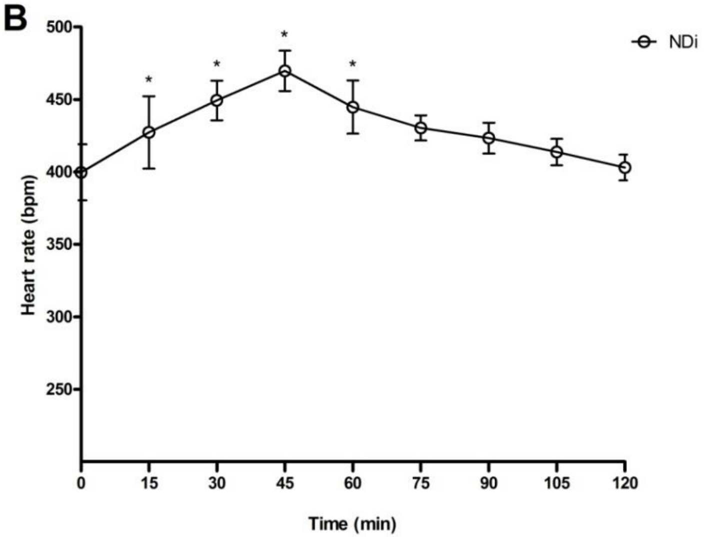

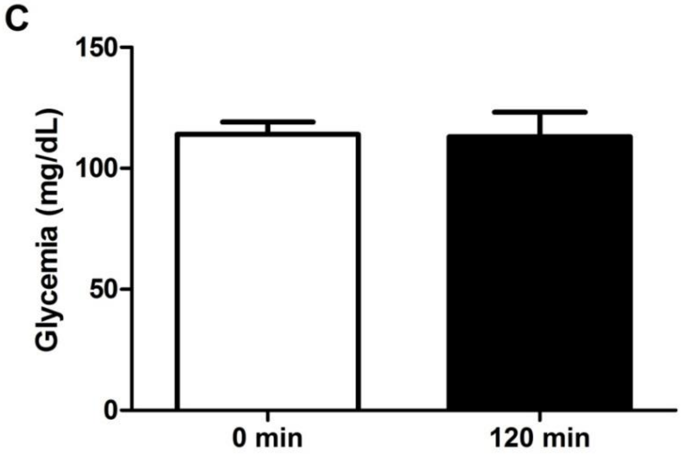

Supplementary Figure 1. Effects of intranasal isoproterenol in non-diabetic rats. Non-diabetic rats were treated with isoproterenol (NDi) as described in Methods, and mean arterial pressure (A), heart rate (B) and blood glucose (C) were measured from zero to 120 min after isoproterenol. Results are means ± SEM of 3 rats; *p<0.05 compared to time zero (basal value), paired Student's t-test.

Supplementary Figure 2

SGLT1 activity in lung alveolar cells of diabetic rats modulates airway surface liquid glucose concentration and bacterial proliferation

Tales Lyra Oliveira, Návylla Candeia Medeiros, Polliane M. Cavalcante-Araújo, Igor Santana Melo, Elaine Fávaro-Pípi, Luciana Alves-Fátima, Antônio Augusto Rocha, Luiz Ricardo Goulart Filho, Ubiratan Fabres Machado, Ruy R Campos, Robinson Sabino-Silva

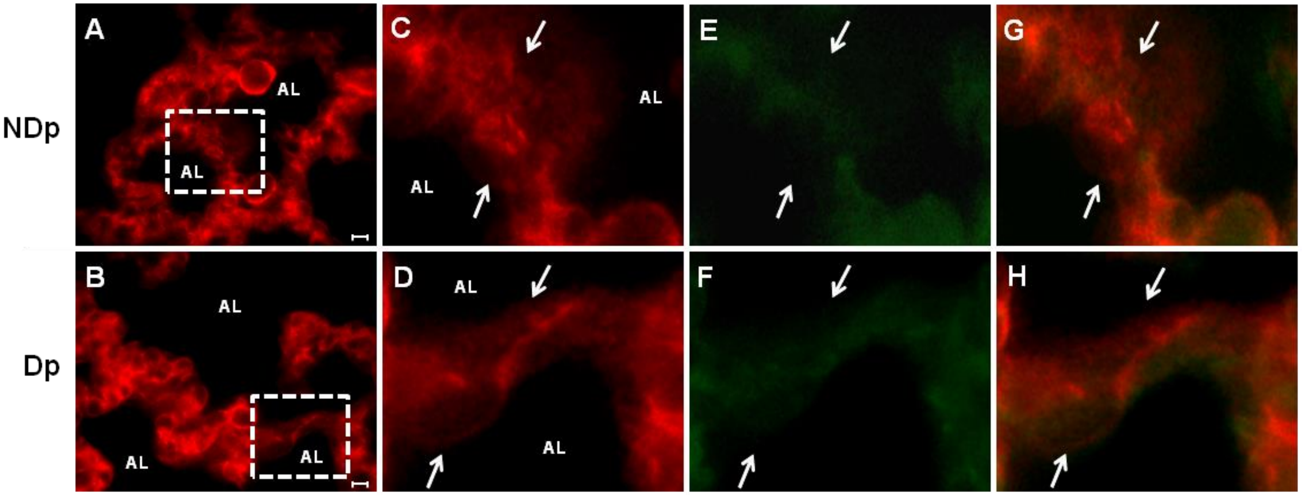

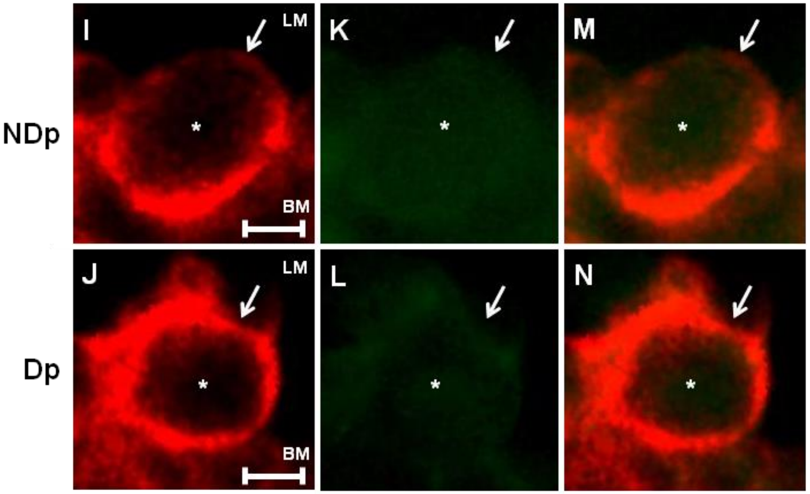

Supplementary Figure 2. Immunodetection of SGLT1 in lung of phlorizin treated rats. In the top, section of pulmonary alveoli from non-diabetic phlorizin (NDp) and diabetic phlorizin (Dp) treated rats. Sections **A and B** were immunostained with anti-F-actin antibody (red). Enclosed boxes showing an alveolar septum, taken with a greater resolution, are presented in the next sections: **(C-D)** F-Actin (red), **(E-F)** SGLT1 (green) and **(G-H)** merged photomicrographs for SGLT1 and F-actin colocalization (yellow to orange). White arrows indicate the luminal membrane; (AL), alveolar lumen. Magnification, x 1000. In the bottom, SGLT1 protein in lung alveolar cells from non-diabetic phlorizin (NDp) and diabetic phlorizin (Dp) treated rats. **(I-J)** F-actin (red), **(K-L)** SGLT1 (green) and **(M-N)** merge. White arrows indicate the luminal membrane, and the asterisks indicate the cytoplasm. (LM), luminal membrane and (BM) basolateral membrane of the pneumocyte. Magnification, x 1000. SGLT1 immunostaining was almost undetectable, supposedly because phlorizin prevents the binding of the anti-SGLT1 antibody. Images are representative of 4-5 animals in each group.
